# Teacher Physical Education Practices and Student Outcomes in a Sample of Middle Schools Participating in the Presidential Youth Fitness Program

**DOI:** 10.5888/pcd16.180627

**Published:** 2019-08-08

**Authors:** Isabela Ribeiro Lucas, Carole Harris, Sarah Lee, Jane Wargo, Seraphine Pitt Barnes, Tina J. Kauh, Ronaldo Iachan

**Affiliations:** 1 ICF, Atlanta, Georgia; 2 Centers for Disease Control and Prevention, Division of Population Health, School Health Branch, Atlanta, Georgia; 3 National Fitness Foundation, Washington, DC; 4 Robert Wood Johnson Foundation, Princeton, New Jersey

## Abstract

Obesity and lack of physical activity among children and adolescents are public health problems in the United States. This Presidential Youth Fitness Program (PYFP) evaluation measured program implementation in 13 middle schools and its effect on physical education practices, student fitness knowledge, and student physical activity and fitness levels. PYFP, a free program with the potential to positively affect student health and fitness outcomes, was designed to improve fitness education practices that are easily integrated into existing physical education programs. We used a 2-group (13 PYFP and 13 comparison schools) quasi-experimental design to collect FitnessGram assessments, accelerometry data, and surveys of students, physical education teachers, and administrators. Although the program was positively associated with student cardiovascular endurance and physical activity gains during the semester, schools underused professional development courses and fitness recognition resources.

**Summary**

**What is already known on this topic?**

Multicomponent school-based physical education (PE) programs can improve children’s health and academic outcomes. An examination of the Presidential Youth Fitness Program (PYFP) on student’s outcomes and PE practices had not been conducted until the present evaluation.

**What is added by this report?**

PYFP was associated with an increase in student aerobic capacity during a semester. PYFP students had significantly higher aerobic capacity at the end of the semester than did comparison students.

**What are the implications for public health practice?**

PYFP is a free and voluntary program that can be implemented in schools across the country and can positively affect PE practices and student outcomes.

## Introduction

Obesity and lack of physical activity among children and adolescents are public health problems in the United States (1,2). The *2018 Physical Activity Guidelines Advisory Committee Scientific Report* confirms a strong association between higher physical activity levels and better health outcomes, including cardiorespiratory and muscular fitness, bone health, and weight status (3). Because school-aged children spend more than half of their waking hours in school (4) and engage in 20% to 30% of their total physical activity at school (5), schools are ideal settings in which to implement interventions to increase physical activity. Multicomponent school-based physical education (PE) programs improve children’s health and academic outcomes (6,7), and a standards-based PE curriculum helps students develop the knowledge and skills needed to achieve and maintain health-enhancing levels of physical activity and fitness (8).

The Presidential Youth Fitness Program (PYFP) was created in 2012 by a public–private partnership between the President’s Council on Sports, Fitness and Nutrition, the Centers for Disease Control and Prevention, the National Fitness Foundation, the Society of Health and Physical Educators, and The Cooper Institute. A process evaluation of PYFP showed positive results (9), but the effectiveness of PYFP on key outcomes was not examined. The objective of this study was to describe findings from a PYFP outcomes evaluation. 

## Purpose and Objectives

PYFP has hypothesized 4 key components to increase health-related fitness and knowledge among students and improve the effectiveness of PE: 1) use of FitnessGram (www.cooperinstitute.org/fitnessgram), a criterion-based fitness assessment that compares student measurements with a set of health-related standards; 2) a focus on fitness education to promote cardiovascular and muscular health; 3) professional development for PE teachers; and 4) recognition for students who achieve Healthy Fitness Zone standards.

We conducted the evaluation in 26 middle schools in the United States from October 2017 through June 2018 with the purpose of addressing the following questions: To what degree was PYFP implemented? Did PYFP implementation lead to integration of fitness education into physical education, improve fitness testing practices, or have a positive effect on PE and physical activity policies, practices, or environments? Did PYFP affect fitness knowledge, physical activity levels, or fitness among students?

## Intervention Approach

On the basis of evidence that fitness assessment and education might influence fitness levels (10), PYFP aims to improve teacher fitness education practices and student knowledge, physical activity levels, and fitness with no cost to schools. PYFP schools included in this evaluation voluntarily applied for a grant from the National Fitness Foundation in 2014 or 2015 to participate in PYFP and, as part of the program, received FitnessGram software licenses, teacher textbooks and online training, and student recognition items.

## Evaluation Methods

The evaluation was based on systems thinking theory, which focuses on linkages and interactions among system components (in this study, components of PYFP) and assesses intended and unintended outcomes (11). We used mixed-methods, a 2-group quasi-experimental design. A power analysis indicated that a sample of 22 schools (11 PYFP schools and 11 control schools) would be appropriate. We used the following data sources:

- surveys of students, PE teachers, and school administrators,
- 2 components of the FitnessGram assessment (the 20-meter Progressive Aerobic Cardiovascular Endurance Run [PACER], designed to assess aerobic capacity, and measurements of height and weight to calculate body mass index [BMI, kg/m^2^]), collected at the beginning (baseline) and end (follow-up) of a PE semester, and
- accelerometry data collected at the beginning (baseline) and end (follow-up) of a PE semester.

We conducted baseline assessments from October 2017 through April 2018 and follow-up assessments from January 2018 through June 2018. ICF’s institutional review board and the US Office of Management and Budget approved the study.

**School selection.** Of 293 public middle schools that received National Fitness Foundation Round 2 (2014–2017) or Round 3 (2015–2018) grants for PYFP, 43 met inclusion criteria (≥50% students receive free or reduced-price lunch; >150 students are enrolled in 6th and 7th grades [combined]) and were eligible to participate. We contacted 28 PYFP schools after our team received approval from their districts; 5 declined, 10 did not respond, and 13 enrolled. PYFP and comparison schools (matched on size, percentage of students receiving free or reduced-price lunch, geography, and racial/ethnic distribution) participated voluntarily. To achieve the target sample, we selected at least 4 PE classes per school. Study participation required parental consent and student assent.

**Participants.** We recruited 4 schools in addition to our targeted 22 schools to prepare for potential attrition, resulting in 26 schools (13 PYFP, 13 comparison) from 9 geographically diverse states (Iowa, Illinois, Indiana, Kentucky, Maine, Michigan, North Carolina, Tennessee, Washington). Forty-eight PE teachers (23 PYFP, 25 comparison) completed online surveys; 2,702 students (1,435 PYFP, 1,267 comparison) completed paper-and-pencil surveys; and 569 students (290 PYFP, 279 comparison) provided accelerometry data. We obtained height and weight measurements for 2,440 students (1,174 PYFP, 1,266 comparison) and PACER measurements for 2,616 (1,375 PYFP, 1,241 comparison) students.

**Data collection.** A trained, designated liaison in each school obtained parental permission, assisted with the logistics of FitnessGram assessments, distributed accelerometers, and implemented student surveys (completed once at the end of the semester). We provided various incentives (eg, fitness equipment, money, gift cards, nonmonetary prizes) to participating schools, liaisons, and students at various levels of participation. PE teachers conducted baseline FitnessGram assessments at PYFP schools, and trained ICF staff conducted them at comparison schools; ICF conducted all follow-up FitnessGram assessments. Students wore ActiGraph accelerometers (model GT3XP-BTLE), positioned on the waist, for 7 days at baseline and 7 days at follow-up.

**Teacher-level data: degree of PYFP implementation and teacher-specific volume of PE.** We measured the degree of PYFP implementation by calculating program dose scores for the following: the proportion of students who received FitnessGram assessments, the number of professional development courses completed by PE teachers (4 were offered), the number of fitness education activities (ie, integration of fitness education into physical education), and use of fitness recognition (certificates awarded to students who score in the Healthy Fitness Zone in at least 5 FitnessGram assessment categories). We developed a scoring algorithm for these data; possible dose scores for each program component ranged from 0 to 4 (for a maximum of 16); higher scores indicate a greater degree of implementation. We measured PE volume for each teacher as the number of PE minutes offered between baseline and follow-up to control for the effect of the program in the regression models.

**School-level data: physical activity/physical education policies, practices, and environment.** We calculated a score for the physical activity/physical education environment from the following items in the PE teacher and administrator surveys: 1) the number of physical activity opportunities outside of PE time, 2) school environmental supports for physical activity/physical education teacher practices, and 3) administrative support. Total possible scores ranged from 0 to 19; higher scores indicate more positive environments. 

**Student-level outcomes.** Outcomes were fitness knowledge (as measured in the student survey), BMI percentile (12), PACER scores (20-meter laps were converted to 1-mile run or walk times to estimate aerobic capacity [maximum oxygen consumption, Vo_2max_]) (13), and intensity of physical activity (time in moderate-to-vigorous physical activity [MVPA]), determined from accelerometer data and child-based cut points (14).

### Analysis

We used Stata version 11 (StataCorp) and SAS version 9.4 (SAS Institute Inc) for all analyses. We calculated descriptive statistics and performed bivariate analysis for school-level, teacher-level, and student-level data. We used multilevel linear models for clustering of students within classrooms for average MVPA (during and outside of PE), Vo_2max_, and BMI percentile. The regression models included students with complete baseline and follow-up data for each outcome: MVPA (n = 387), Vo_2max_ (n = 1,985), and BMI (n = 1,783). Because baseline Vo_2max_ differed between groups, we analyzed follow-up scores by using a group interaction term. We found no group differences at baseline for BMI and MVPA, so we examined change from baseline to follow-up. On the basis of a sensitivity analysis, we included in MVPA analyses data from students with accelerometry data for 3 or more days of 8 hours per day (55% of all observations). We excluded from BMI analyses students whose BMI was greater than 70 (n = 7) or whose height decreased from baseline to follow-up (n = 361). Vo_2max _analyses excluded PE classes with documented deviations from the measurement protocol (9 classes; 210 students). 

## Results

Student demographic characteristics did not differ significantly between groups for school enrollment, percentage of students who receive free or reduced-price lunch, or race (Non-Hispanic black and non-Hispanic white), but PYFP schools had a significantly greater percentage of Hispanic students than comparison schools (11% vs 7%; *P* = .01) (Table).

**Table:** Summary of Findings in Study of Teacher Physical Education Practices and Student Outcomes in a Sample of Middle Schools Participating in the Presidential Youth Fitness Program, 2017–2018

Characteristic	PYFP Schools (n = 13)	Comparison Schools (n = 13)	*P* Value^a^
**Demographic Characteristics of Schools**			
Total school enrollment, mean no. of students	459	553	.22
Students who receive free or reduced-price lunch, %	64	60	.29
Non-Hispanic white, %	75	75	.90
Non-Hispanic black, %	9	9	.50
Hispanic, %	11	7	.01
**Physical Education Implementation^b^**			
**Degree of implementation, as measured by program dose scores, mean (range)**			
No. of teachers who completed online survey	23	25	—
Overall program dose, no. of points scored from 0–16	10.4 (5–15)	—	—
FitnessGram assessments, no. of points scored from 0–4	3.9 (3–4)	—	—
Integration of fitness education into physical education, no. of points scored from 0–4	2.9 (1–4)	—	—
Fitness recognition, no. of points scored from 0–4	2.4 (0–4)	—	—
Professional development courses completed by physical education teachers, no. of points scored from 0–4	1.2 (0–4)	—	—
**Integration of fitness education into physical education^c^**			
Time devoted to fitness education during physical education increased with PYFP	9 of 23 (39%)	—	—
Physical education teacher allocates >50% of physical education time to fitness education	11 of 23 (48%)	12 of 25 (48%)	—
**Fitness testing practices**			
Physical education teacher required students to keep a log of physical activity outside of physical education class	10 of 23 (43%)	4 of 25 (16%)	.36
Physical education teacher provided students with feedback on individuals student physical activity plans	12 of 23 (52%)	8 of 25 (32%)	.45
**Physical education/physical education policies, practices, and environment**			
Administrators reporting that PYFP had a positive effect on school climate	12 of 13 (92%)	—	—
Administrators agreeing that PYFP added value to physical education and physical activity programs by improving PE quality	11 of 13 (85%)	—	—
Physical education teachers reporting increased opportunities for physical activity breaks during school	5 of 23 (22%)	—	—
Physical education teachers reporting increased physical activity during physical education	4 of 23 (17%)	—	—
**Student Outcomes^d^**			
**Fitness knowledge**			
No. of students answering survey questions on knowledge	1,435	1,267	—
Exercise ≥5 days per week for good health, %	70	70	.32
Exercise ≥60 min per day for good health, %	59	59	.34
Learned how to be fit in their physical education classes, %	81	83	.48
Learned about setting goals in physical education to improve fitness scores, %	69	72	.14
**Student BMI percentile**			
No. of students for whom height and weight data were available	792	1,188	—
Baseline assessment, mean (SE)	71.4 (1.0)	69.1 (0.8)	.09
Follow-up assessment, mean (SE)	71.4 (1.0)	69.8 (0.8)	.22
Change between baseline and follow-up, mean (SE)	0.03 (0.32)	0.67 (0.24)	.11
**Student Vo_2max_ e**			
No. of students for whom data were available	951	1,239	—
Baseline assessment, mean (SE)	41.8 (0.2)	41.0 (0.2)	<.001
Follow-up assessment, mean (SE)	42.1 (0.2)	42.2 (0.2)	.64
Change between baseline and follow-up, mean (SE)	0.26 (0.1)	1.19 (0.09)	<.001

Abbreviation: —, not applicable; BMI, body mass index; PE, physical education; PYFP, Presidential Youth Fitness Program; SE, standard error.

a *P* values determined by Levene test for equality of variances for demographic characteristics; by Pearson χ^2^ test for fitness testing practices; by Wald test for fitness knowledge; and by 2-sample *t *test for student BMI percentile and student Vo_2max_.

b Teacher-level variables; online surveys were completed by teachers once during semester.

c Fitness education covers such concepts as the importance of health-related fitness and physical activity for good health.

d Student-level variables; paper-and-pencil surveys were completed by students once during semester; BMI and Vo_2max_ were measured at beginning and end of semester.

e Determined by bivariate analysis of PACER scores; 20-m laps were converted to 1-mile run/walk times to estimate aerobic capacity (maximum oxygen consumption, Vo_2max_) (12). Vo_2max_ is measured in mL of oxygen used in 1 minute per kg of body weight (mL/kg/min).

**Degree of PYFP implementation.** Of the program dose scores, the highest scores were received for FitnessGram assessments (3.9 of 4 points), followed by integration of fitness into PE (2.9 of 4 points). The lowest score was for completion of professional development courses (1.2 of 4 points); only 6 teachers completed 2 or more courses. Almost 40% of teachers reported time devoted to fitness education increased after PYFP implementation, and PYFP teachers reported greater use of student physical activity logs (44% vs 16%) and individual feedback on students’ physical activity plans (52% vs 32%) than comparison teachers.

Most administrators (92%) reported that PYFP had a positive effect on school climate; 85% agreed that PYFP added value to PE, physical activity programs, and students by improving PE quality. However, only 22% of PE teachers reported that PYFP had increased opportunities for physical activity breaks during school, and only 17% indicated that physical activity increased during PE.

**Student outcomes.** Student surveys showed no significant differences in knowledge between groups. Most students in both groups knew the importance of exercising 5 days or more per week, knew that 60 minutes of daily exercise is needed for good health, learned how to be fit in PE classes, and learned about setting fitness goals to improve fitness scores.

Student BMI percentiles were not significantly different between groups at baseline or follow-up, and change from baseline to follow-up was not significantly different between groups. MVPA levels were not significantly different between groups at baseline or follow-up (Figure), but the MVPA of PYFP students increased significantly more than the MVPA of comparison students (*P* = .04). In multivariate models, changes in MVPA and BMI from baseline to follow-up did not differ significantly by group after adjusting for age, sex, teacher-specific volume of PE, baseline values, and physical activity/physical education environment score. Younger students (*P* = .03) and students who were offered higher volumes (frequency and length) of PE (*P* =.03) had significantly lower BMI than older students and those with lower PE volumes. No predictors were significantly associated with the MVPA model.

**Figure:**
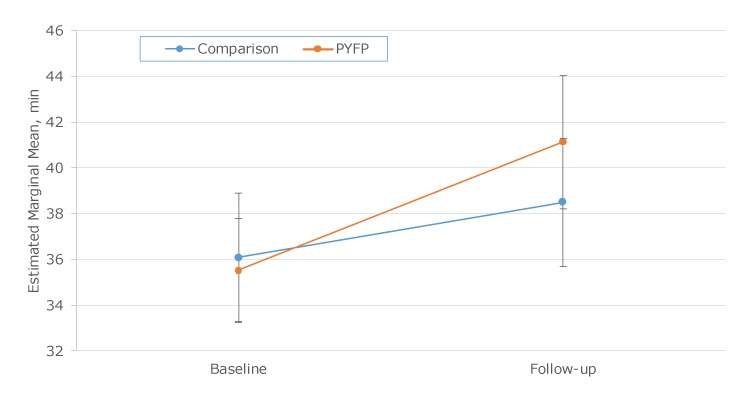
Minutes of daily moderate-to-vigorous physical activity levels at baseline and follow-up, by group, in an evaluation of student outcomes in a sample of middle schools participating in the Presidential Youth Fitness Program (PYFP), 2017–2018. The evaluation comprised 13 PYFP schools and 13 comparison schools. Error bars indicate 95% confidence intervals.

**Table:** 

Study Group	Baseline	Follow-Up
Comparison group	36.1 (0-126.7)	38.5 (0-130.5)
PYFP	35.5 (7.4–115.0)	41.1 (7.3–107.7)

In the bivariate analysis, baseline Vo_2max_ was modestly but significantly higher among PYFP students than comparison students, whereas change in Vo_2max_ from baseline to follow-up was significantly higher among comparison students. After we adjusted for student age, student sex, teacher-specific PE volume, baseline Vo_2max_, and physical activity/physical education environment, the regression model for Vo_2max_ at follow-up showed significant group effects, with higher scores at follow-up for PYFP students than comparison students (*P* < .001), but no group differences for the change-over-time model. Being younger (*P *= .01) and having higher baseline Vo_2max_ (*P* < .001) were significant predictors for follow-up Vo_2max_.

## Implications for Public Health

This evaluation was the first to assess the effect of PYFP on student health and fitness and to use comparison schools. Findings indicated school administrators and teachers strongly supported PYFP and attributed substantial improvements in PE courses and PE/PA environments to the program. Moreover, the positive associations between PYFP and student cardiovascular endurance at follow-up provided evidence for the health benefits of the program. Because PYFP’s components are consistent with the recommendations and evidence described in the National Physical Activity Plan (15) and the Comprehensive School Physical Activity Program (16), and they involve little or no cost to participating schools, PYFP should be considered a strong and practical strategy to improve student physical activity levels. Interestingly, degree of PYFP implementation was not significantly associated with student outcomes, and professional development courses and fitness recognition resources were underused, which suggests more research is needed to determine the amount of training required for teachers and the role of student recognition in promoting student fitness achievements.

Our study has several limitations. We did not randomly assign schools to PYFP or comparison conditions. PYFP schools had voluntarily started the program 2 or 3 years before the evaluation, and the evaluation was designed to examine PYFP *as implemented.* Thus, a selection bias may have been present. The use of matched comparison schools and statistical controls was intended to minimize the influence of factors known to affect student fitness (eg, race/ethnicity, sex, age), but they might not have eliminated the influence of known and unknown factors. In addition, our study was retrospective, so reports by school personnel might have been influenced by memory bias. Lack of random assignment and the retrospective design preclude the ability to determine cause and effect. Because of the time required to obtain approvals and recruit schools, the study period was limited to 1 semester. A longer study period might have produced different findings.

PYFP is a free program with the potential to positively affect student health and fitness outcomes. Strategies to support greater and more consistent use of PYFP resources, such as professional development, and program enhancements to address implementation barriers should be considered.
